# Developing and aging: A tale of two stages

**DOI:** 10.1111/cns.13264

**Published:** 2019-11-12

**Authors:** Hanna Lu

**Affiliations:** ^1^ Department of Psychiatry The Chinese University of Hong Kong Hong Kong China; ^2^ Guangdong Engineering Technology Research Center for Translational Medicine of Mental Disorders Guangzhou China; ^3^ The Affiliated Brain Hospital of Guangzhou Medical University Guangzhou China

To the editors,

We read with great interest of Kang et al's study to investigate the safety and effects of repetitive transcranial magnetic stimulation (rTMS) on brain activity, behavior, and cognitive function in children with low‐functioning autism.^1^ Kang and colleagues addressed the preintervention brain functional connectivity through off‐line electroencephalography (EEG), which offers an imaging marker to guide and predict the efficacy of rTMS. Considering the high variability in autism spectrum disorder (ASD), the process of diagnosing and grouping the subtypes of ASD is complicated. Based on the core symptoms of autism, the additional “communication” domain that outlined impairments in verbal (ie, language), which could be assessed by cognitive tests. Compared to high‐functioning autism, the low‐functioning autism is usually compounded by presence of intellectual disability and psychological dysfunctions, of which the brain networks tend to be more delicate and disorganized.^2^ Thus, treatment options for individuals with low‐functioning autism are quite limited.

Device‐based interventions, such as noninvasive brain stimulation (NIBS), have been applied in adult neurology and psychiatry for many years. Recently, some researchers have applied NIBS to children and adolescents. Unlike pharmacological treatment, transcranial magnetic stimulation (TMS) is unique technique in the clinical neuroscience that has very specific spatial resolution and mechanism of action. There are convincing data supporting that TMS can focus on a single domain whose network function is relatively well‐known and has been shown to be responsible to rTMS. There is much excitement about the promise of this novel and advanced technology; meanwhile, there is also a clear and urgent need when conducting TMS in the field of pediatric psychiatric and developmental disorders.

Despite the therapeutic advances of rTMS, the translation to the clinic poses a number of critical complexities and challenges, including: (a) Chronological age: The mean age of ASD is around 6‐8 years, of which the brain is still developing. Based on the knowledge we learned from aging brain, the dynamic changes within a short period, such as half a year, can be recognized from functional and structural MRI, which highlights the need to combine individual MRI data; (b) Scalp‐to‐cortex distance (SCD): As highlighted in the NIBS guidelines,^3^ SCD, as a key parameter of neuroimaging and brain stimulation, could significantly influence the focality and strength of electric field^4^; (c) Heterogeneity: The large variability in the therapeutic effect of NIBS studies may be caused by the inter‐individual differences of neurophysiological, cognitive, and morphometric features. For example, the large clinical and neurophysiological (ie, MEP) variability in children with ASD and the overlapping symptoms with other pediatric psychiatric disorders, researchers, and clinicians need to thoroughly characterize and when possible stratify participants based not only on their diagnosis, but also the cognitive domain being investigated; (d) Region‐specific: Of particular relevance of TMS, neurodevelopmental disorders have been associated with the abnormal activity within the cortical hubs of specific brain network, such as dorsolateral prefrontal cortex (DLPFC), posterior parietal cortex (PPC), and supplementary motor area (SMA).^2^ Although DLPFC is the region of interest, network‐based TMS will bring more informative evidence of stimulation‐induced effects on intellectual ability in autism; (e) Safety: Any technique that involves delivering magnetic stimulation to the brain must consider safety. There are reports summarized the pain, headache, and even seizures during the rTMS treatment of brain stimulation.^5^ However, the standardized protocol to monitor and record the potential side effect is still lacking.

Collectively, a way forward in the application of NIBS is to tailor the treatment to individual patients based on their chronological or morphometric profiles. Of note, the differences between responders and nonresponders to NIBS may be caused by the inter‐individual region‐specific morphometric measures, such as cortical thickness, scalp‐to‐cortex distance, and cortical folding. Combing morphometric features of the targeted region will be a recommendation for improving clinical NIBS practice in children, adolescents, and seniors.

## CONFLICT OF INTEREST

The authors declare no conflict of interest.
